# Structural modifications to pregnane neurosteroids alter inhibition of LPS/Lipid A binding at the MD-2 activation site within the TLR4 signaling complex

**DOI:** 10.3389/fimmu.2025.1632891

**Published:** 2025-09-04

**Authors:** Alejandro G. Lopez, Venkat R. Chirasani, Irina Balan, A. Leslie Morrow

**Affiliations:** ^1^ Department of Biochemistry and Biophysics, University of North Carolina at Chapel Hill, Chapel Hill, NC, United States; ^2^ Bowles Center for Alcohol Studies, School of Medicine, University of North Carolina at Chapel Hill, Chapel Hill, NC, United States; ^3^ R. L. Juliano Structural Bioinformatics Core, School of Medicine, University of North Carolina at Chapel Hill, Chapel Hill, NC, United States; ^4^ Department of Psychiatry, School of Medicine, University of North Carolina at Chapel Hill, Chapel Hill, NC, United States; ^5^ Department of Pharmacology, School of Medicine, University of North Carolina at Chapel Hill, Chapel Hill, NC, United States

**Keywords:** TLR4, allopregnanolone, neurosteroids, MD-2, molecular dynamics, surface plasmon resonance, structure activity relationships

## Abstract

Neurosteroids have emerged as promising candidates for treatment for neuroinflammatory diseases, distinct from their classical GABAergic effects. We previously demonstrated that 3α,5α-THP inhibits binding of the Lipid A moiety of lipopolysaccharide to the Toll-like receptor 4 (TLR4): Myeloid Differentiation factor 2 (MD-2) protein complex with nanomolar affinity, suggesting that this mechanism may underlie its ability to inhibit TLR4 signal activation in macrophages and brain. This study investigates the structure activity relationships (SAR) for this action of pregnane neurosteroids, focusing on their interactions with MD-2. Through a combination of molecular docking, surface plasmon resonance, and molecular dynamics simulations, we evaluated how modifications to the A, C, and D rings of neurosteroids influence their interactions with MD-2, including binding affinity, orientation, and conformation. The data reveal that hydrophobic interactions, particularly involving PHE151, may be key to neurosteroid binding to MD-2, and that D ring modification may alter the competitive inhibition of Lipid A binding and subsequent TLR4 activation by pregnane steroids. Furthermore, the prototypical neurosteroids 3α,5α-THP and progesterone demonstrated deeper MD-2 pocket binding and greater MD-2 stabilization, while SGE 516 induced MD-2 flexibility and weaker competitive inhibition compared to 3α,5α-THP. These insights establish a unique structural and mechanistic basis for the immunomodulatory activity of these neurosteroids and offer a novel conceptual framework for future rational design of therapeutics targeting TLR4-mediated neuroinflammation.

## 1 Introduction

Endogenous neurosteroids have emerged as significant candidates for the treatment of neurological and psychiatric disorders, with a recent focus on those disorders characterized by inflammatory neuroimmune signaling in the brain. Synthesized primarily in neurons, certain neurosteroids illicit inhibitory effects on neurotransmission by allosteric modulation of gamma-aminobutyric acid type A (GABA_A_) receptors, providing sedative, anxiolytic, and anti-convulsant properties (1–3). However, their anti-inflammatory properties are multifaceted and distinct from their GABAergic modulation, giving neurosteroids a wide range of therapeutic implications for the treatment of both systemic and neuroimmune inflammatory disorders.

Studies have recognized decreased levels of the pregnane class of neurosteroids in disorders such as depression (4), post-traumatic stress disorder (PTSD) (5) and alcohol use disorder (AUD) (6), that led to clinical and preclinical work showing therapeutic properties in brain disorders such as AUD, traumatic brain injury (TBI), multiple sclerosis, and Alzheimer’s disease (AD) (6, 7). Notably, the recent Food and Drug Administration (FDA) approval of Brexanolone for the treatment of post-partum depression (PPD), an intravenously administered formulation of [3α,5α]-3-hydroxypregnan-20-one (3α,5α-THP, Allopregnanolone), has displayed its effectiveness on neuroimmune associated brain disorders. Specifically, Brexanolone infusion decreased inflammatory cytokine expression, lipopolysaccharide (LPS)-induced Toll-like receptor 4 (TLR4) activation and imiquimod (IMQ)-induced toll-like receptor 7 (TLR7) activation in whole blood cell lysates of patients (8). Importantly, these effects predicted clinical improvement in the patients, demonstrating the impact of the anti-inflammatory actions of this neurosteroid.

3α,5α-THP and pregnenolone inhibit the expression and activation of key TLR4 signaling proteins such as tumor necrosis factor receptor-associated factor 6 (TRAF6), growth factor beta-activated kinase 1 (TAK1), nuclear factor kappa-B (NF-κB), mitogen-activated protein kinases (MAPK) and decrease the expression of downstream proinflammatory mediators such as interleukin 6 (IL-6), high mobility group box 1 protein (HMGB1), interleukin 1 beta (IL-1β), monocyte chemoattractant protein-1 (MCP-1), and tumor necrosis factor alpha (TNF-α) (9–11). Both steroids appear to disrupt TLR4 binding to Myeloid Differentiation 2 (MD-2) to prevent TLR4 pathway activation. In addition, progesterone has been observed to reduce TLR4 activation through indirect phosphorylation of NF-κB, decreasing expression and translocation to the nucleus (12) and through decreasing mRNA expression of TLR4, MyD88 and NF-κB (13).

Progesterone, 3α,5α-THP, and pregnenolone share structural similarities at their D rings; however, neurosteroids such as 3α,5α-THDOC and SGE 516, although GABAergic, do not possess the same ability to effectively inhibit pro-inflammatory signaling (11). The structures of 3α,5α-THDOC and SGE 516 differ from the other pregnane steroids at their D rings, where modifications of single hydroxyl and triazole groups are held at C-21, respectively. In human macrophages, these modifications were deleterious in inhibiting TLR4 signaling (11).

3α,5α-THP inhibition of TLR4 signaling has been shown to involve several components of the cascade. Specifically, 3α,5α-THP inhibits protein–protein interactions between TLR4 and key signaling partners such as Myeloid Differentiation 2 (MD-2), Myeloid Differentiation Primary Response 88 (MyD88), Toll-Interleukin-1 Receptor (TIR) domain-containing adaptor protein (TIRAP), and the GABA_A_ α2 subunit protein (9, 10, 14). Among these interactions, recent work has highlighted that 3α,5α-THP inhibits the binding of the endotoxin component of TLR4 agonist lipopolysaccharide (LPS), Lipid A, to TLR4 co-receptor MD-2 (14). The structural basis of LPS/Lipid A binding to the TLR4:MD-2 complex has been characterized, with key interacting amino acids mapped (15, 16). The TLR4-MD-2-LPS complex contains two major functional interfaces: the primary interface, where MD-2 binds to a single TLR4 molecule, and the dimerization interface, located on the opposite face of MD-2, which facilitates the dimerization of TLR4 via interactions mediated by MD-2 and LPS.

Prior work has shown that MD-2 adopts a β-cup fold formed by anti-parallel β sheets, creating a hydrophobic pocket at the dimerization interface where Lipid A binds, thus forming hydrophobic bonds with 5 of its 6 lipid chains. The sixth chain interacts with the outer surface of MD-2 further stabilizing the dimerization interface and enabling TLR4 activation. The role of MD-2 is critical for TLR4 activation in immune cells such B lymphocytes, macrophages, dendritic cells (17) and in the brain (18). Moreover, splice variants of MD-2 have been shown to diminish the agonist properties of LPS, reducing TLR4 activation and downstream cytokine production (19, 20).

Based on the collective evidence, we hypothesized that structural modifications, particularly at the D-ring, of pregnane neurosteroids would alter their ability to bind MD-2 and inhibit LPS/Lipid A-induced TLR4 activation. To test this, we conducted a SAR analysis of selected pregnane neurosteroids, including 3α,5α-THP, progesterone, 3α,5α-THDOC, and SGE 516, using molecular docking, surface plasmon resonance (SPR), and molecular dynamics simulations. These methods allowed us to focus specifically on interactions within the LPS/Lipid A binding pocket of MD-2.

## 2 Materials and methods

### 2.1 Molecular docking

#### 2.1.1 Protein and ligand preparation

The three-dimensional crystal structure of the human TLR4 in complex with MD-2 was retrieved from the RCSB Protein Data Bank (PDB ID: 3FXI) (15). Structural visualization and preliminary preparation of the receptor complex were performed using BIOVIA Discovery Studio Visualizer software (Version 21.1.0.20298, Dassault Systems, San Diego, CA, USA).

The ligand structures of [3α,5α]-3-hydroxypregnan-20-one (3α,5α-THP) and 14 additional neurosteroids selected based on modifications to the A, C, or D rings relative to 3α,5α-THP, including progesterone, 3α,5α-THDOC, and SGE 516, were retrieved from the Zinc20 chemical database (https://zinc20.docking.org/). All compounds were converted to PDBQT format using the Open Babel GUI (21) to ensure compatibility with docking tools.

#### 2.1.2 Preparation of docking inputs

The TLR4:MD-2 complex was prepared by adding polar hydrogens, assignment of Gasteiger partial charges, and conversion of atom types to AutoDock Vina-compatible format using MGL Tools (Version 1.5.7). Flexible torsions were assigned to the ligand to allow rotational freedom during docking. The TLR4:MD-2 complex was treated as a rigid receptor during this process. Molecular docking simulations were conducted using AutoDock Vina (Version 1.2.0), which enabled both ligand conformational sampling and estimation of binding affinity (22).

#### 2.1.3 Docking protocol and analysis

The grid box was designed to comprehensively cover the MD-2 binding cavity, with dimensions set to 54 × 40 × 58 Å³, with the grid box center positioned at x = −4.658, y = 2.529, and z = −2.961. For each docking run, ten distinct binding poses were generated. These poses were ranked based on their predicted binding affinity (expressed as binding energy in kcal/mol). A total of 10 docking poses were generated for each compound, and were subsequently ranked based on binding energy, allowing for the selection of poses with a maximum energy difference of 3 kcal/mol and a minimum root-mean-square deviation (RMSD) threshold of 1.0 Å. Post-docking analysis was carried out using Discovery Studio Visualizer to characterize protein-ligand interactions, including hydrogen bonding, hydrophobic contacts, and key amino acid residues involved in binding stabilization.

### 2.2 Surface plasmon resonance

#### 2.2.1 Ligand immobilization

SPR data were collected using OpenSPR-XT instrument (Nicoya, Canada). Recombinant human MD-2 (R&D Systems Inc., Minneapolis, MN, USA, # 1787-MD-050/CF) was covalently immobilized on High Sensitivity Carboxyl Sensors (Nicoya, Kitchener, ON, Canada) and 1:1 N-hydroxysuccinimide (NHS)/1-ethyl-3-(3-dimethylaminopropyl) carbodiimide hydrochloride (EDC) reactive coupling reactivity. Preconcentrated MD-2 (10 mM Acetate Buffer, pH 5.5) was injected over an activated carboxyl sensor at 30 µg/mL with a flow rate of 20 µL/min (1× PBS, 0.1% Tween-20), resulting in capturing roughly 4000 RU. The remaining active carboxyl sites were blocked using 1 M Ethanolamine (pH 8.5, Nicoya) for 300 s. Binding assays were performed using 1× PBS (0.05% Tween-20).

#### 2.2.2 Competition assay

Lipid A (AdipoGen Life Sciences, San Diego, CA, USA, IAX-100-004-M001) was prepared using the manufacturer’s protocol. Briefly, a stock solution of 1 mg/mL was heated to 40°C for 5 min before diluting to a working concentration in running buffer to mitigate Lipid A aggregation. Lipid A was prepared in running buffer (1× PBS + 0.05% Tween-20) to a final concentration of 25 µM, serving as the primary binding analyte. Neurosteroids of interest were serially diluted in dimethyl sulfoxide (DMSO) and spiked with each Lipid A well at varying concentrations to assess their competitive binding against Lipid A. The assays were conducted at 25°C to ensure thermal stability in interaction kinetics. All reported response curves were double referenced and subtracted from the reference flow cell and buffer blanks, which served as negative controls to account for non-specific binding and baseline drift. Binding data were analyzed using TraceDrawer SPR evaluation software (Version 1.9.2, Uppsala, Sweden) and fitted with a 1:1 binding model. The concentration of Lipid A was chosen based on the ability to provide an adequate response (RU) and neurosteroid concentrations were selected after screening a range of concentrations that fit within physiologically relevant doses (2-fold dilutions; 313 nM-10 µM). Neurosteroids were serially diluted using two-fold dilutions and added to Lipid A just before beginning the assay and were tested in triplicate (n=3).

### 2.3 Molecular dynamics simulations

Molecular dynamics simulations were performed to study the interactions between MD-2 and the reference neurosteroid 3α,5α-THP, as well as comparative simulations with 14 additional pregnane neurosteroids including progesterone, 3α,5α-THDOC, and SGE 516, using the GROMACS 2021.5 (23) package and the CHARMM36 force field (24).

#### 2.3.1 System Preparation

To prepare the simulation system, the MD-2 structure was solvated in a triclinic box containing explicit TIP3P water molecules. To mimic physiological conditions, Na^+^ and Cl^-^ ions were added to achieve electroneutrality and an ionic strength of 150 mM.

#### 2.3.2 Energy minimization and equilibration

Prior to production runs, the system underwent energy minimization using the steepest descent algorithm to eliminate unfavorable steric clashes. Subsequently, a two-phase equilibration process was performed: an initial 5 ns equilibration under constant volume and temperature (NVT) ensemble, followed by 10 ns under constant pressure and temperature (NPT) ensemble. During these equilibration phases, positional restraints were applied to heavy atoms of the protein-ligand complex.

#### 2.3.3 Simulation parameters

The production simulations were conducted for 1000 ns with temperature maintained at 303.15 K using the velocity-rescaling (v-rescale) thermostat and pressure held at 1 bar using the C-rescale barostat (25). Bond constraints involving hydrogen atoms were applied using the LINCS algorithm (26), allowing for an integration time step of 2 fs. Long-range electrostatics were treated using the Particle Mesh Ewald (PME) method (27), and both van der Waals and electrostatic interactions employed a cutoff distance of 1.2 nm.

### 2.4 Molecular dynamics trajectory analysis

Comprehensive post-simulation analysis was conducted using GROMACS tools built-in tools along with custom Python scripts. Root-mean-square deviation (RMSD) and root-mean-square fluctuation (RMSF) analyses were performed to assess the stability and flexibility of MD-2 over the simulation period. Additionally, non-bonded contacts between the neurosteroids and MD-2 were thoroughly analyzed. To quantify the non-bonded contacts between 3α,5α-THP and MD-2, the *gmx mindist* tool was employed, applying a distance cutoff of 5 Å. Protein–ligand interaction profiles were further analyzed using LigPlot+ (28), which generated schematic diagrams of hydrogen bonds and hydrophobic contacts. Trajectory movies were generated using VMD (29) to illustrate the dynamic behavior, conformational changes, and binding stability of the MD-2 and neurosteroid complexes.

## 3 Results

### 3.1 Neurosteroids screened to evaluate SARs

**Figure 1:**
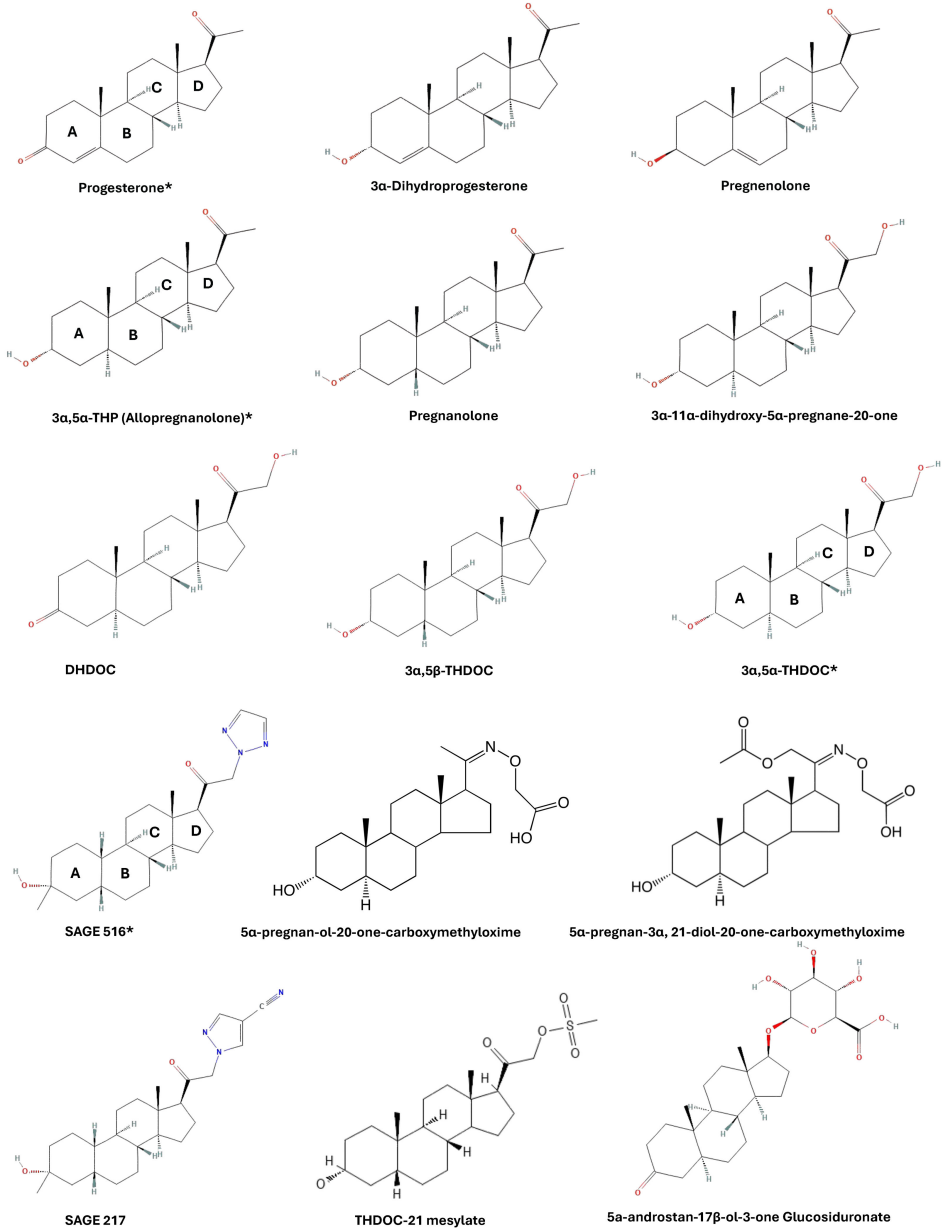
Neurosteroid structures used for molecular docking screen. Compounds were selected based on modifications to A, C, and D rings. For illustration purposes, the compounds are fixed with their A rings left and D rings right and are organized by molecular mass (low to high).

The goal of this screen was to identify the structural requirements for the reference neurosteroid 3α,5α-THP to inhibit LPS/lipid-A binding to the MD-2 co-receptor of TLR4. A set of structurally related pregnane neurosteroids were selected for comparison based on modifications to the A, C, and D rings of 3α,5α-THP. The modifications are categorized as follows: 1) A ring modification: pregnenolone, progesterone, 3α-Dihydroprogesterone, and pregnanolone, 2) C ring modifications: 3α-11α-dihydroxy-5alpha-pregnane-20-one, 5α-pregnan-3α,21-diol-20-one-carboxymethyloxime, 5α-pregnan-OL-20-one-carboxymethyloxime, 5α-androstan-17β -OL-3-one glucosiduronate, and 3) D ring modifications: 3α,5α-THDOC, 3α,5β-THDOC, THDOC-21 mesylate, SGE 516, SGE 217, 5α-Dihydrocorticosterone (DHDOC). The structures of these compounds are provided in **Figure 1**, organized by molecular mass and shown as 2D representations of 3D structures used in the molecular docking screen. The rings of 3α,5α-THP are labeled (A-D) and 3α,5α-THDOC, progesterone, and SGE 516 are marked with asterisks.

### 3.2 Molecular docking analysis of neurosteroid screen

**Figure 2:**
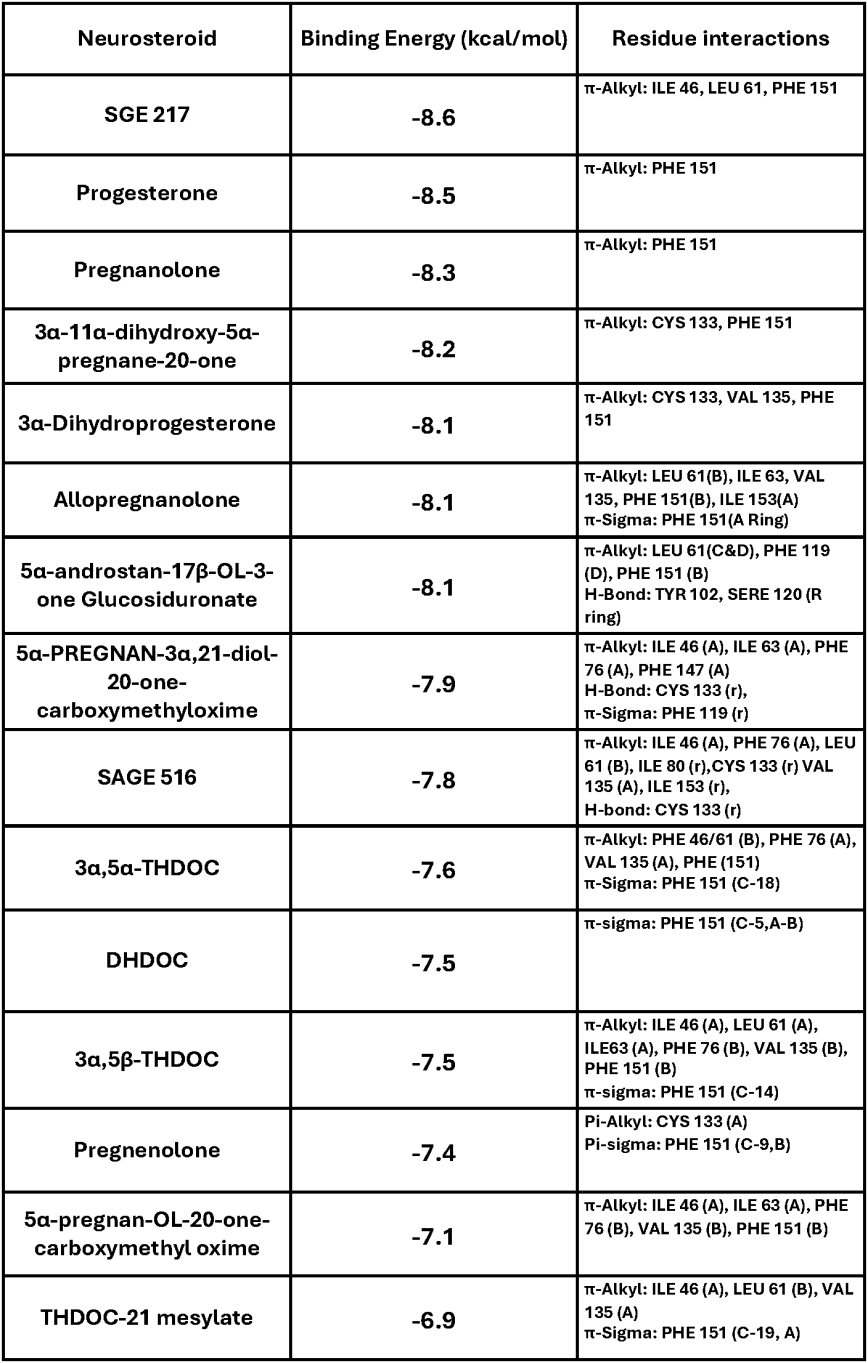
Molecular docking screen of neurosteroids to MD-2. Each neurosteroid was screened against MD-2. This table provides data from the docking screen, organized by their binding energy (kcal/mol). Top poses of each neurosteroid were investigated to provide the residue interactions and bond types observed in molecular docking analysis.

These fifteen neurosteroids were screened against the hydrophobic pocket of MD-2, the known binding site for LPS/Lipid A. The docking screen provided detailed interactions of each neurosteroid and their binding scores, with a range of -8.6 to -6.9 kcal/mol (**Figure 2**). The analysis of residue interactions across the compounds reveals key residues involved in binding, with PHE 151 emerging as the most frequent contact, observed in 13 of 15 neurosteroids, except 5α-pregnan-3α,21-diol-20-one-carboxymethyloxime, SGE 516, and pregnenolone. This residue likely plays a critical role in stabilizing neurosteroid binding, facilitating both π-alkyl and π-sigma bonds.

Following PHE 151, ILE 46, CYS 133, and VAL 135 were each observed in six or more neurosteroid complexes. Specifically:

- ILE 46: SGE 217, 5α-pregnan-3α,21-diol-20-one-carboxymethyloxime, SGE 516, 3α,5α-THDOC, 3α,5β-THDOC, 5α-pregnan-OL-20-one-carboxymethyl oxime, THDOC-21 mesylate.
- CYS 133: 3α-11α-dihydroxy-5α-pregnane-20-one, 3α-dihydroprogesterone, 5α-pregnan-3α,21-diol-20-one-carboxymethyloxime, SGE 516, pregnenolone.
- VAL 135: 3α-dihydroprogesterone, 3α,5α-THP, SGE 516, 3α,5α-THDOC, 3α,5β-THDOC, 5α-pregnan-OL-20-one-carboxymethyl oxime, THDOC-21 mesylate.

These residues are implicated in LPS-dependent TLR4 activation, as mutations in VAL 135 have been shown to disrupt CD14-mediatiated LPS transfer to MD-2 (30).

Additionally, LEU 61 was identified in the following neurosteroid interactions: SGE 217, 3α,5α-THP, 5α-androstan-17β-OL-3-one glucosiduronate, SGE 516, 3α,5α-THDOC, 3α,5β-THDOC, and THDOC-21 mesylate. PHE 76 was involved in: 3α,5α-THP, 5α-pregnan-3α,21-diol-20-one-carboxymethyloxime, SGE 516, 3α,5α-THDOC, 3α,5β-THDOC, and 5α-pregnan-OL-20-one-carboxymethyl oxime. These findings further highlighting the importance of hydrophobic interactions within MD-2.

### 3.3 Modifications to D ring alter competitive binding to MD-2

**Figure 3:**
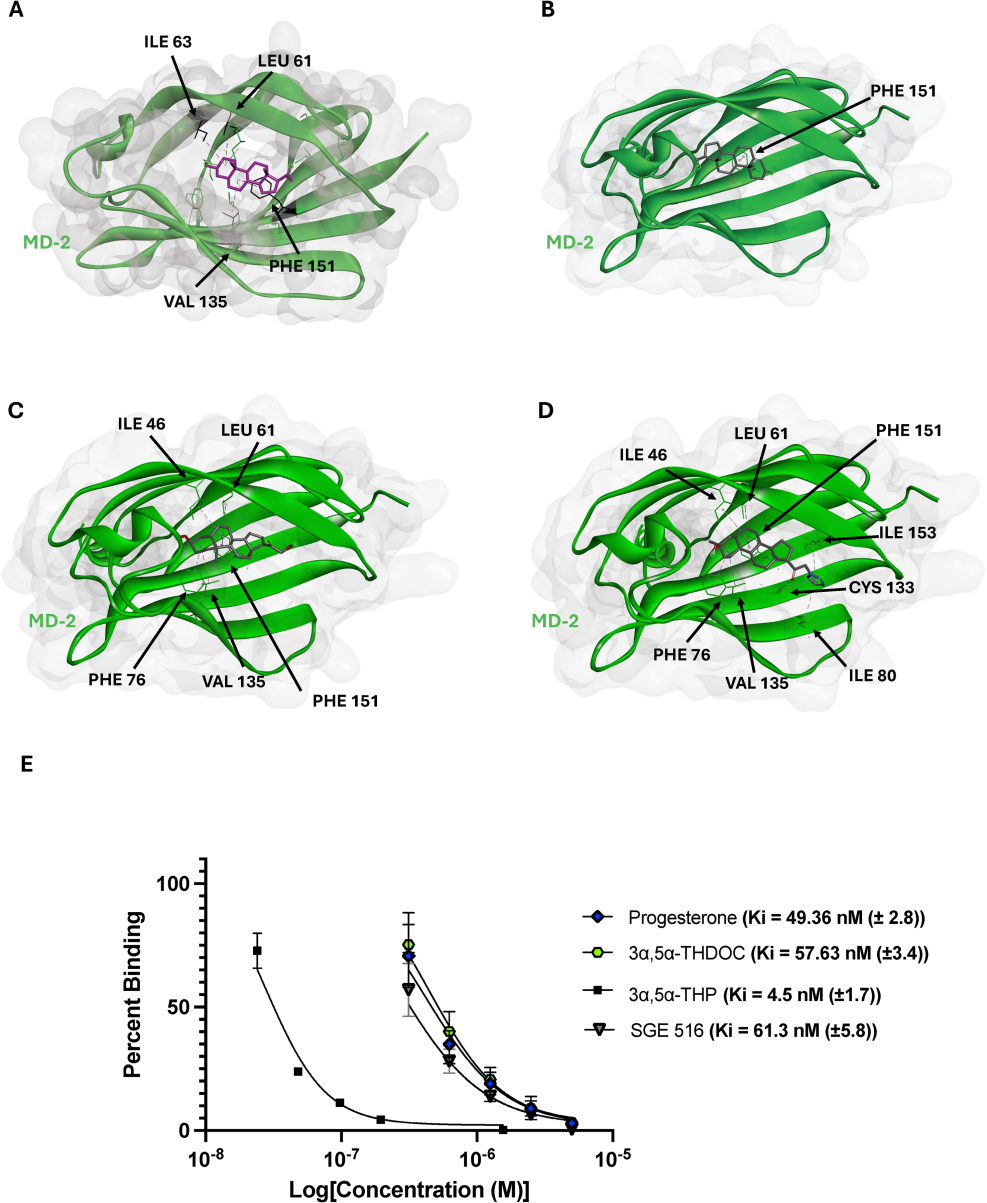
Top selected neurosteroids inhibit lipid A binding to MD-2. Molecular docking images of 3α,5α-THP **(A)**, progesterone **(B)**, 3α,5α-THDOC **(C)**, and SGE 516 **(D)**. These neurosteroids display reasonable docking within MD-2, with interacting residues such as PHE 151, ILE 46, LEU 61, and CYS 133. The inhibition constants were obtained from top scoring neurosteroids and are as follows 3α,5α-THP (4.5 ± 1.7 nM), progesterone (49.36 ± 2.8), 3α,5α-THDOC (57.63 ± 3.4 nM), and SGE 516 (61.3 ± 5.8 nM) **(E)**.

As we have previously demonstrated the ability of 3α,5α-THP to competitively bind the LPS/Lipid A binding site in MD-2, we tested several additional neurosteroids. These compounds were selected based on their molecular docking binding energy scores, commercial availability, *in vivo and in vitro* data availability, and ring modification type and location. Progesterone (−8.5 kcal/mol), 3α,5α-THDOC (−7.6 kcal/mol), and SGE 516 (−7.8 kcal/mol) were among the top-scoring compounds. **Figures 3A-D** shows 3α,5α-THP, progesterone, 3α,5α-THDOC and SGE 516 docked into the MD-2 hydrophobic pocket and the corresponding interacting residues.

Interestingly, we observed that 3α,5α-THP, 3α,5α-THDOC and progesterone each formed a π-alkyl bond with PHE 151 through their B rings. In contrast, SGE 516 did not interact with PHE 151 but still showed favorable interactions with other hydrophobic residues, highlighting the role of LEU 61, VAL 135, ILE 46, and CYS 133 in MD-2 ligand stabilization. Although similar interactions were seen, the docked orientations varied: 3α,5α-THP and progesterone inserted their D rings deeper into the MD-2 cavity, whereas 3α,5α-THDOC and SGE 516 oriented their D rings toward the MD-2 “opening”. To further evaluate whether these structural differences correlated with biological activity, we conducted SPR competition assays to measure inhibition constants (Ki) for MD-2:Lipid-A binding. The previously reported Ki for 3α,5α-THP was 4.5 ± 1.7 nM (14). 3α,5α-THP, progesterone, 3α,5α-THDOC and SGE 516 each demonstrated a strong competitive binding profile, each showing a dose-dependent reduction of lipid A binding to MD-2. Progesterone, 3α,5α-THDOC, and SGE 516 showed Ki values of 49.36 ± 2.8 nM), 57.63 ± 3.4 nM, and 61.3 ± 5.8 nM, respectively (**Figure 3E**). These values are approximately tenfold higher than for 3α,5α-THP, suggesting that D ring modifications in 3α,5α-THDOC and SGE 516 may reduce their inhibitory potency on MD-2:Lipid-A binding and consequently, on TLR4 signaling.

### 3.4 Distinct interaction and flexibility profiles of neurosteroids in MD-2 binding

**Figure 4:**
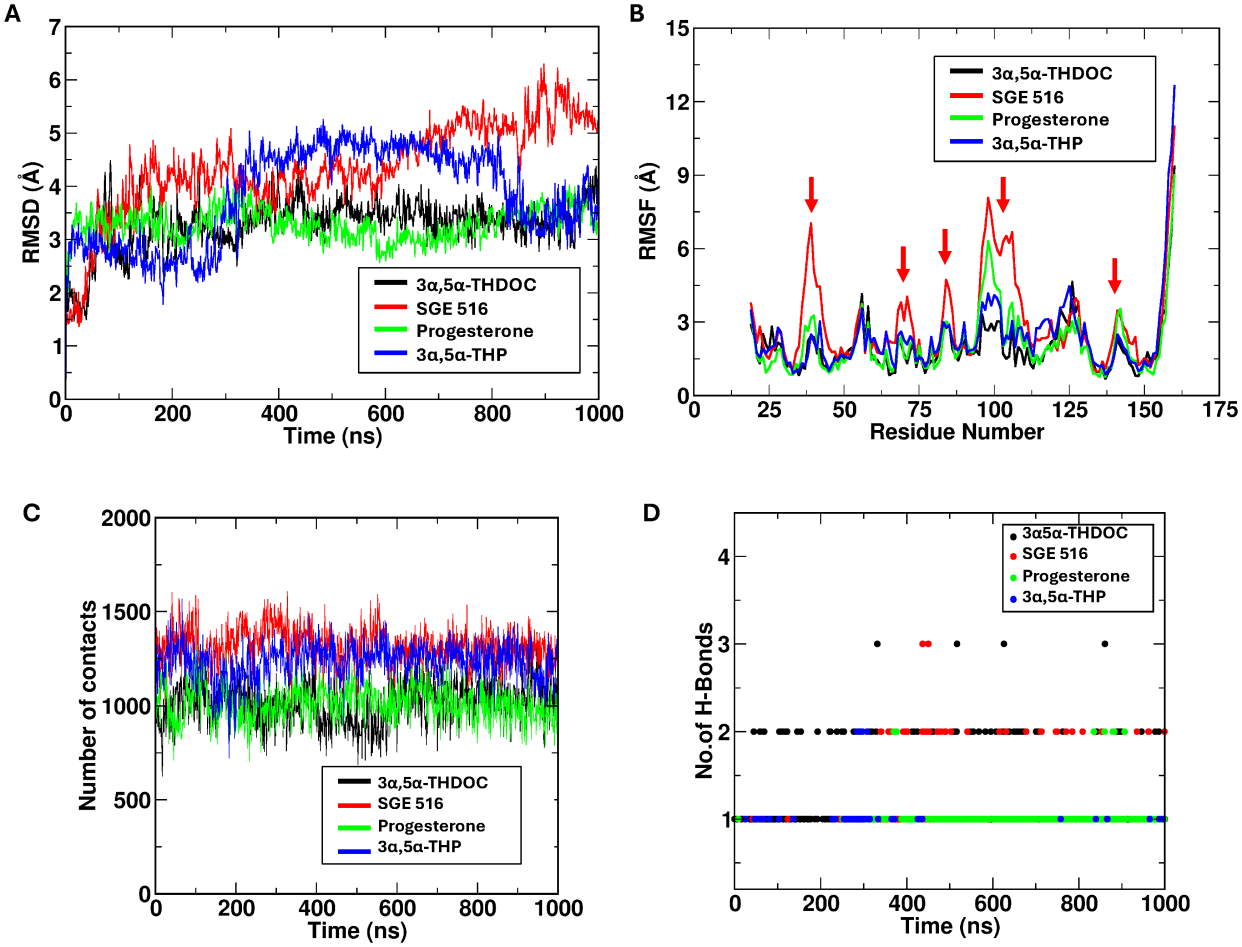
Progesterone, 3α,5α-THP and 3α,5α-THDOC binding stabilize MD-2 structure. This figure shows data obtained from molecular dynamics (40) simulations. SGE 516 demonstrates higher number of contacts in comparison to progesterone, 3α,5α-THDOC **(A)**, and 3α,5α-THP **(B)**. RMSD and RMSF depict the ability of progesterone, 3α,5α-THDOC, and 3α,5α-THP to stabilize MD-2 structure, while SGE 516 produces greater conformational flexibility when bound to MD-2. SGE 516 and 3α,5α-THP displayed higher number of contacts during the simulation in contrast to 3α,5α-THDOC and progesterone **(C)**. SGE 516 and 3α,5α-THDOC formed higher numbers of hydrogen bonds which may correlate to the C-21 modifications of each neurosteroid **(D)**.

Molecular dynamic simulations were performed for three neurosteroids — SGE 516, progesterone, and 3α,5α-THDOC based on prior molecular docking results. The simulations revealed notable differences in the dynamic behavior of MD-2 in the presence of these ligands. Among them, SGE 516 exhibited the highest number of interactions with MD-2, surpassing those observed for progesterone, 3α,5α-THP, and 3α,5α-THDOC. This suggests that SGE 516 forms a more extensive interaction network within the binding pocket (**Figures 4A, B**). This increased interaction density was accompanied by elevated number of contacts and hydrogen bonds throughout the simulation (**Figures 4C, D**). Higher RMSD and RMSF values in the SGE 516–MD-2 complex indicate that this ligand may enhance the conformational flexibility of MD-2, likely due to the broader conformational sampling enabled by its interaction profile. These findings suggests that SGE 516 may both stabilize the binding pocket and induce functional flexibility in MD-2, potentially modulating its activity.

In contrast, 3α,5α-THDOC and progesterone elicited dynamic profiles similar to those of 3α,5α-THP (14). Both ligands formed a comparable number of interactions with MD-2, without inducing notable changes in RMSD or RMSF relative to the apo state (**Figures 4A, B**). The protein exhibited relatively stable dynamics in their presence, with minimal fluctuations in the binding pocket. This suggests that binding of 3α,5α-THDOC or progesterone may favor a more rigid or stabilized MD-2 conformation, leading to fewer conformational transitions compared to the SGE 516 bound system. **Table 1** summarizes the approaches and key data compiled in this study.

**Table 1:** Summary of molecular docking, SPR binding affinity, and Molecular Dynamics data for selected neurosteroids to MD-2.

Neurosteroid	Inhibition Constant (Ki)	Compound orientation	Binding energy (kcal/mol)	Residue interactions	MD simulation # of contacts	MD simulation MD-2 flexibility
3α,5α-THP	4.5 nM ± (1.7)	D ring towards MD-2 cavity	-8.1	π-Alkyl: LEU 61(B),PHE 151(B), ILE 153(A) π-Sigma: PHE 151(A Ring)	~1300	Highest MD-2 rigidity
Progesterone	49.36 nM (± 2.8)	D ring towards MD-2 cavity	-8.5	π-Alkyl: PHE 151	~1000	Increased MD-2 Rigidity
3α,5α-THDOC	57.63 nM ( ± 3.4)	D ring towards MD-2 opening	-7.6	π-Alkyl: PHE 46/61 (B), PHE 76 (A), VAL 135 (A), PHE (151) π-Sigma: PHE 151 (C-18)	~1000	Increased MD-2 Rigidity
SAGE 516	61.3 nM ( ± 5.8)	D ring towards MD-2 opening	-7.8	π-Alkyl: ILE 46 (A), PHE 76 (A), LEU 61 (B), ILE 80 (r), CYS 133 (r) VAL 135 (A), ILE 153 (r), H-bond: CYS 133 (r)	~1400	Least rigidity, highest flexibility

**Table 1** shows the comparison across molecular docking, SPR, and molecular dynamic data. All compounds docked with D ring either towards the MD-2 cavity or opening. Inhibition constants were measured SPR competition assay, and molecular docking binding energies (kcal/mol) are reported with key interacting MD-2 residues. Additionally provide are molecular dynamics average number of contacts and visual assessment of MD-2 flexibility. Compounds with the higher rigidity, such as 3α,5α-THP, 3α,5α-THDOC and progesterone showed higher affinity and few conformational fluctuations, whereas SGE 516 exhibited the greatest flexibility despite a high number of contacts.

## 4 Discussion

This study provides some of the first insight into the SAR of neurosteroids and their interactions with the MD-2 hydrophobic pocket, a critical site in LPS-dependent TLR4 activation. Using molecular docking, molecular dynamics, and surface plasmon resonance, we investigated how the structural modifications to the A, C, and D rings of neurosteroids affect their binding properties and influence the functionality of MD-2. These findings reveal that numerous neurosteroids exhibit binding to MD-2 and provide binding energy scores ranging between -8.6 kcal/mol to -6.9 kcal/mol, with PHE 151 emerging as a key residue involved across most screened compounds. Other residues including ILE 46, CYS 133, VAL 135, LEU 61, and PHE 76, may further contribute to the hydrophobic interactions that underlie the interactions within MD-2. Prior research examining the binding characteristics of the semisynthetic compound Artemisinin, has described similar interactions between the compound and PHE 151, ILE 46, and CYS 133 as being critical for ligand binding to MD-2 (31). In BV2 microglial cells, Artemisinin drastically decreased the expression of IL-1β, Nitric Oxide (NO), and TNF-α, similar to the effects of 3α,5α-THP in human macrophages and alcohol preferring (P rat) brain (10). These amino acids appear crucial in the hydrophobic and π interactions that underlie the binding of each neurosteroid and represent key features of the target binding site.

Molecular docking analysis revealed that D ring modifications may affect the orientation of the neurosteroid within the MD-2 pocket. Notably, 3α,5α-THP and progesterone bind deeper within the MD-2 cavity, whereas 3α,5α-THDOC and SGE 516 form D ring bonds closer to the MD-2 opening. This spatial difference in binding orientation may significantly influence the functional outcomes of neurosteroid binding, as interactions at the MD-2 opening resemble those observed for TLR4 agonists, including Neoseptin-3 and LPS. A similar phenomenon has been observed for the TLR4 synthetic agonist Neoseptin-3, where its phenol rings bind preferentially at the outer entrance of MD-2 (32). The agonistic properties are attributed to the positioning of Neoseptin-3 binding at the interface of TLR4:MD-2:LPS. This interface is situated at the opening of MD-2 and plays a crucial role in the binding of LPS, subsequently facilitating the activation of the TLR4:MD-2 complex. To further enhance the findings of these docking studies, the implementation of *in vitro* and *in silico* mutational screens of the MD-2 pocket and structural mapping of neurosteroid-bound MD-2 are necessary.

SPR assays further corroborated our computational findings, demonstrating that progesterone, 3α,5α-THDOC, and SGE 516 all inhibited lipid A binding to MD-2 with nanomolar inhibition constants. Progesterone, 3α,5α-THDOC, and SGE 516 exhibited Ki values of 49.36 nM, 57.63 nM, and 61.3 nM, respectively, though their affinities were about 10-fold weaker than that of 3α,5α-THP. Although all three steroids demonstrated competitive binding properties, it appears that D ring modifications in 3α,5α-THDOC and SGE 516 may hinder their abilities to inhibit TLR4 signaling effectively. The weaker affinities of progesterone, 3α,5α-THDOC, and SGE 516 relative to 3α,5α-THP may also reflect reduced binding stability or fewer key contacts in the MD-2 pocket, particularly in the case of progesterone, which primarily engages PHE 151. Progesterone may exhibit weaker inhibitory activity because it binds MD-2 primarily via a single interaction, whereas 3α,5α-THP, 3α,5α-THDOC, and SGE 516 form additional stabilizing contacts with residues like LEU 61, VAL 135, ILE 153, and CYS 133, which may enhance their binding affinity and inhibitory effects. The reduced efficacy of 3α,5α-THDOC and SGE 516 in SPR assays may correspond with their previously observed partial inhibition of LPS-induced TLR4 signaling in human monocyte-derived macrophages, in contrast to the stronger inhibitory effect of 3α,5α-THP (11).

The functional role of MD-2 in TLR4 signaling relies on a structurally stable hydrophobic pocket that anchors LPS, which allows the protein to accommodate diverse ligands and mediate TLR4 dimerization and activation (15, 16). Studies have found that the β-cup formation of MD-2 changes in the presence of different binding partners, and it is more rigid when bound to agonists such as LPS (33). The observed rigidity of MD-2 is essential for ligand affinity, which promotes proper TLR4 signaling. Interestingly, the rigid state of MD-2 has been observed through other molecular dynamic studies using known MD-2:LPS inhibitors, suggesting that MD-2 rigidity also impacts the ability of neurosteroids to bind to MD-2 (34).

Molecular dynamics simulations provided further insight into how neurosteroids modulate MD-2 conformational dynamics. SGE 516 induced greater conformational flexibility of MD-2 and formed a more promiscuous binding network than that of 3α,5α-THDOC, progesterone, and 3α,5α-THP. This phenomenon may be attributed to the incorporation of a triazole group into the D ring, which can facilitate both hydrogen bonding and hydrophobic interactions (35). In contrast, 3α,5α-THDOC and progesterone stabilized the MD-2 structure, promoting a relatively rigid conformation. These distinct effects suggest that SGE 516 may exert its functional influence by enhancing the dynamic flexibility of MD-2, whereas 3α,5α-THDOC and progesterone stabilized MD-2, promoting a relatively rigid conformation.

Additionally, similar results have been observed with nicotine and its structurally similar metabolite, cotinine. Molecular dynamics studies of the two bindings within the MD-2 pocket revealed that nicotine maintained structural rigidity in comparison to cotinine (36). This difference in measured RMSD values correlated to *in vivo* studies, where cellular thermal shift assays demonstrated nicotine increased the rigidity of MD-2 in comparison to its metabolite. Therefore, the increase in rigidity of MD-2 may prevent its interaction with LPS, which is required for TLR4 signal activation. In contrast, a study investigating a potential TLR4 antagonist ACT001 found the opposite effect on MD-2, where binding of the target compound increased MD-2 flexibility, while maintaining its ability to inhibit TLR4 signaling (37). The differences observed of structural changes of MD-2 hint’s that the binding modality of each compound may vary depending on interacting amino acids and warrants future studies. Taken together, these findings highlight the distinct effects of different neurosteroids on the conformational dynamics of MD-2. The plots of RMSD and RMSF and the contact analysis provide further insights into the dynamics of these protein-ligand interactions, supporting these observations.

Although this work provides the foundation for understanding the SAR of neurosteroid inhibition of TLR4 signaling through the binding within the MD-2 activation site, the study is limited to *in silico* and SPR binding techniques. To further assess the binding of neurosteroids to MD-2, more in depth structural analysis is required. Structural studies such as X-ray crystallography or cryo-electron microscopy would ultimately reveal the interactions occurring between neurosteroids to MD-2, and their interactions to the TLR4:MD-2:LPS complex. It should also be noted that, due to the low number of proteins that can be incorporated into the experimental procedures, the physiological relevance of multiprotein binding events may be limited. For instance, lipid A and MD-2 are just two components of a multiprotein interaction involving TLR4 and LPS binding, which overlooks significant accessory proteins like CD14. The binding of LPS to CD14 is necessary for LPS-dependent TLR4 signaling as CD14 enhances the binding stability of LPS to the TLR4:MD-2 complex (16). Although previously discussed, our experimental procedures negate the dimerization interfaces in TLR4:MD-2:LPS binding. These dimerization interfaces are integral for multiple reasons: 1) the stability of TLR4 dimerization, 2) the stability of TLR4:MD-2 binding, and 3) the binding and activation of TLR4 by LPS (15, 16). The lack of these interfaces may explain the differences observed in the inhibition kinetics conducted in this study.

To address these concerns, future studies should utilize mutational screening to evaluate differences in binding energies amongst key MD-2 residues found through molecular docking, followed by site-directed mutagenesis of these residues and SPR binding studies. This work could then be expanded to include additional neurosteroids demonstrating favorable binding energies within MD-2. Since our molecular docking-based SAR screen was limited to 15 compounds, a larger screening of all neurosteroid-like compounds, including the additional 11 compounds listed in this study could provide further SAR information and potentially uncover even more potent TLR4:MD-2 inhibitors.

Future SPR studies are needed on a broader set of neurosteroids, especially those with larger D ring modifications to further validate the idea that D-ring modifications affect inhibition of lipid A binding to MD-2 and consequently the activation of TLR4 signaling. Additionally, we previously demonstrated that 3α,5α-THP inhibits MyD88 to TIRAP (14), inferring that neurosteroids such as 3α,5α-THP may have multiple inhibitory sites that merit further investigation.

Collectively, these findings underscore the structural features and dynamic behaviors that underpin neurosteroid interactions with MD-2. Here, we provide a framework for understanding how structural modifications to neurosteroids can influence their ability to modulate TLR4-mediated proinflammatory signaling. Further investigation of these critical SARs will aid in the rational design of neurosteroid-based therapeutics targeting MD-2 and other TLRs, with the potential to address a range of neuroinflammatory and immune-associated disorders. In recent years, neurosteroids have been FDA approved for post-partum depression such as Brexanolone (3, 8) and Zuranolone (38). Additionally, Ganaxolone was also approved for the treatment of seizures associated with CDKL5 deficiency (39) thus providing further evidence for the feasibility of neurosteroids as treatment options. Moreover, the SAR insights provided here could inform the development of novel neurosteroid derivatives that selectively modulate immune signaling while minimizing effects on the central nervous system, a desirable property for treating systemic inflammatory diseases.

## Data Availability

The datasets generated for this study, including molecular docking input/output files and molecular dynamics simulation input/output files, are available in the Zenodo repository at https://doi.org/10.5281/zenodo.16997794. Full MD trajectories are available from the corresponding author upon reasonable request.
