# Supplementary figures and images for: Structural modifications to pregnane neurosteroids alter inhibition of LPS/Lipid A binding at the MD-2 activation site within the TLR4 signaling complex
Source: Front Immunol. 2025 Sep 4;16:1632891. doi: 10.3389/fimmu.2025.1632891 (PMC12443555; doi:10.3389/fimmu.2025.1632891)

## Slide 1
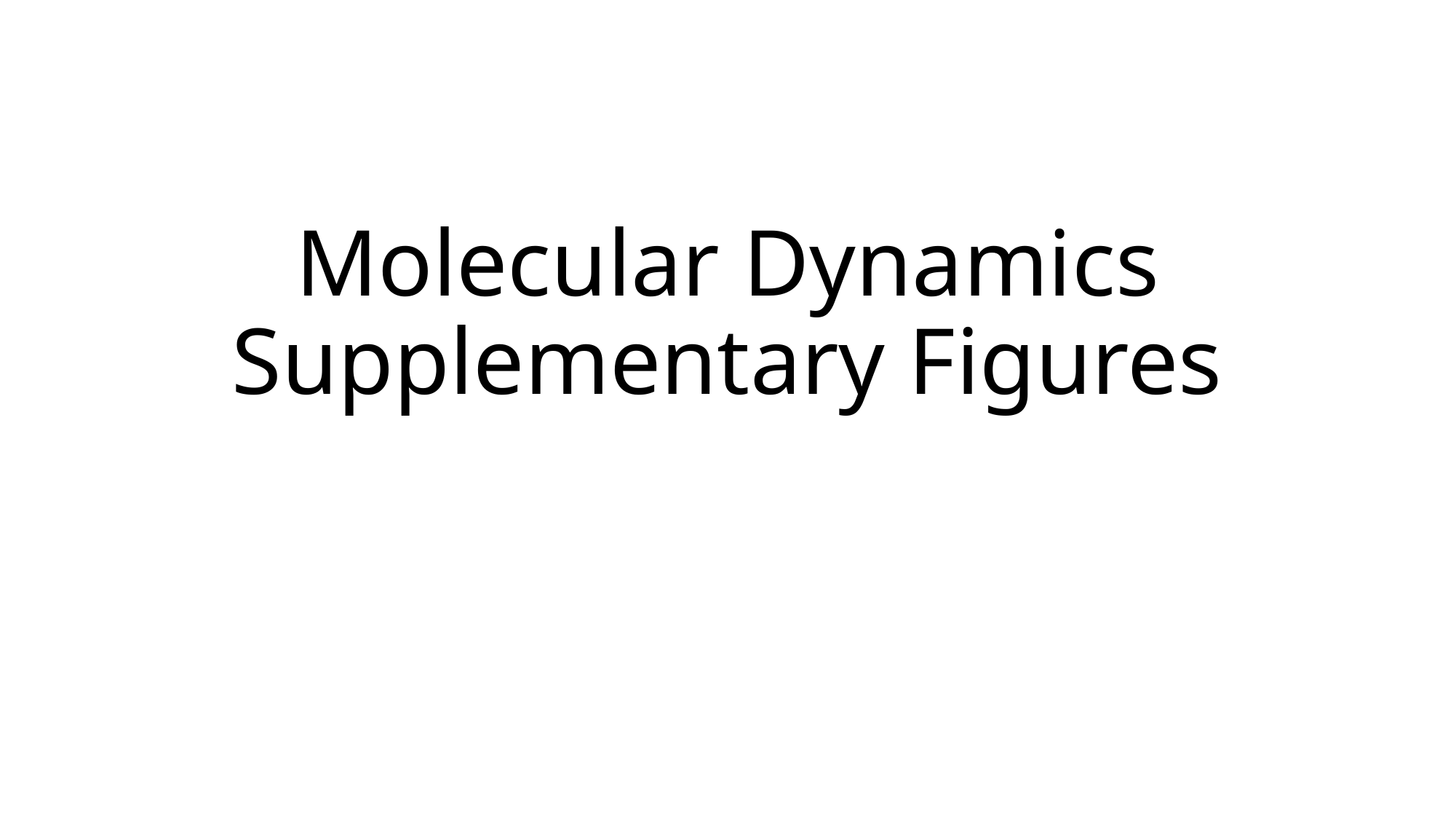

# Molecular Dynamics Supplementary Figures

## Slide 2
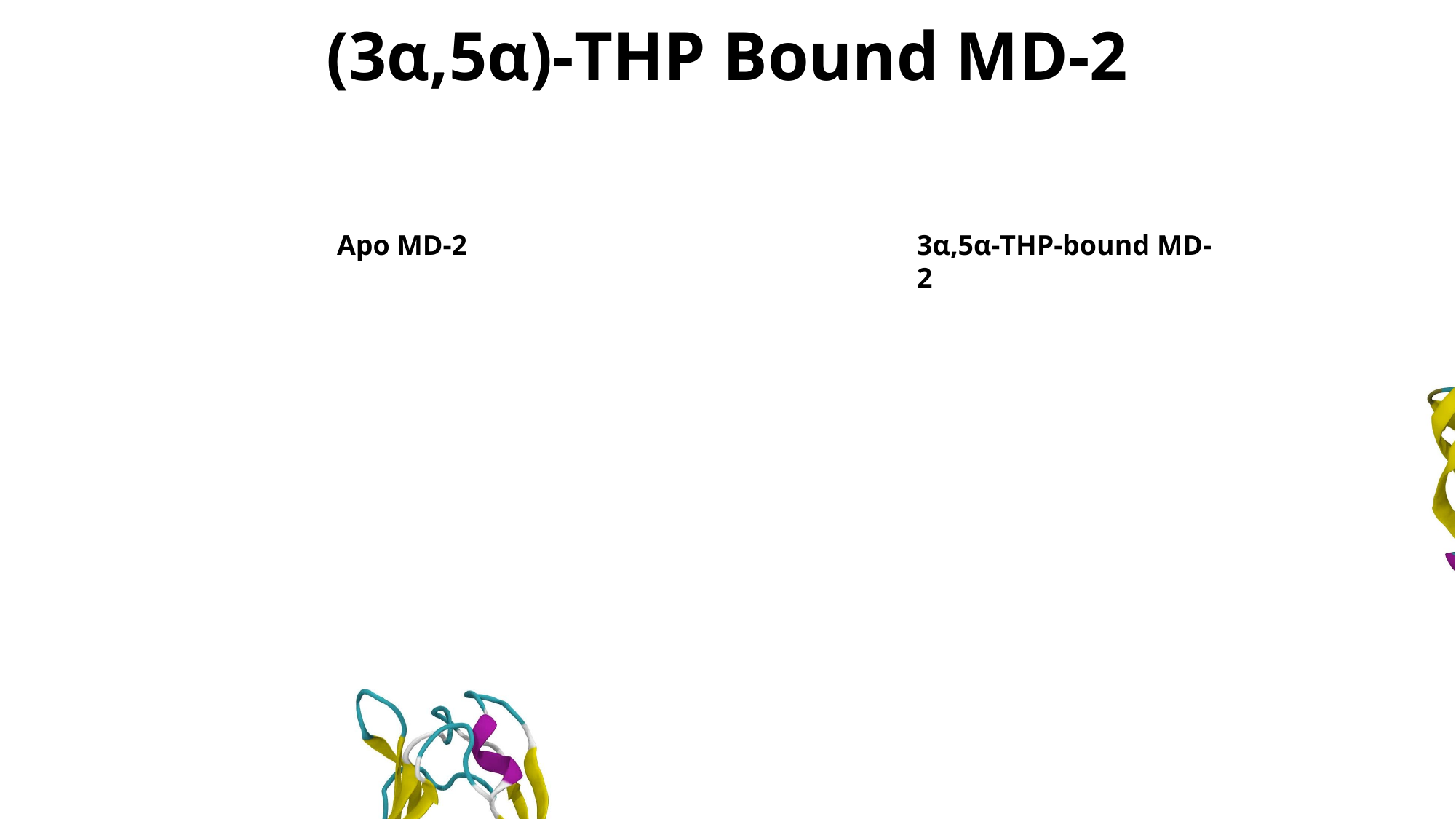

# (3α,5α)-THP Bound MD-2
Apo MD-2
3α,5α-THP-bound MD-2

Supplement: Supplementary file 1 [file Presentation1.pptx]

## Slide 1
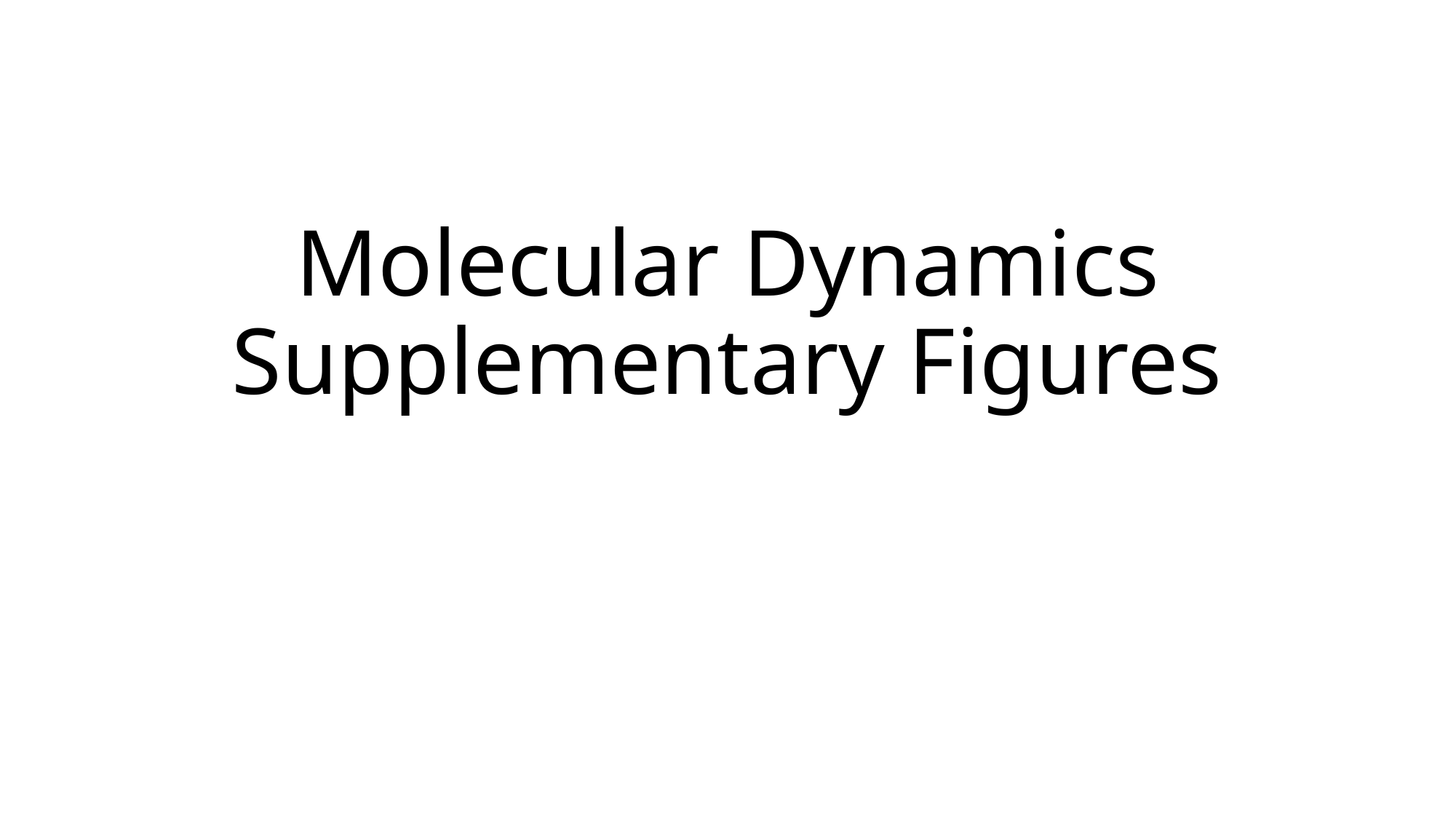

# Molecular Dynamics Supplementary Figures

## Slide 2
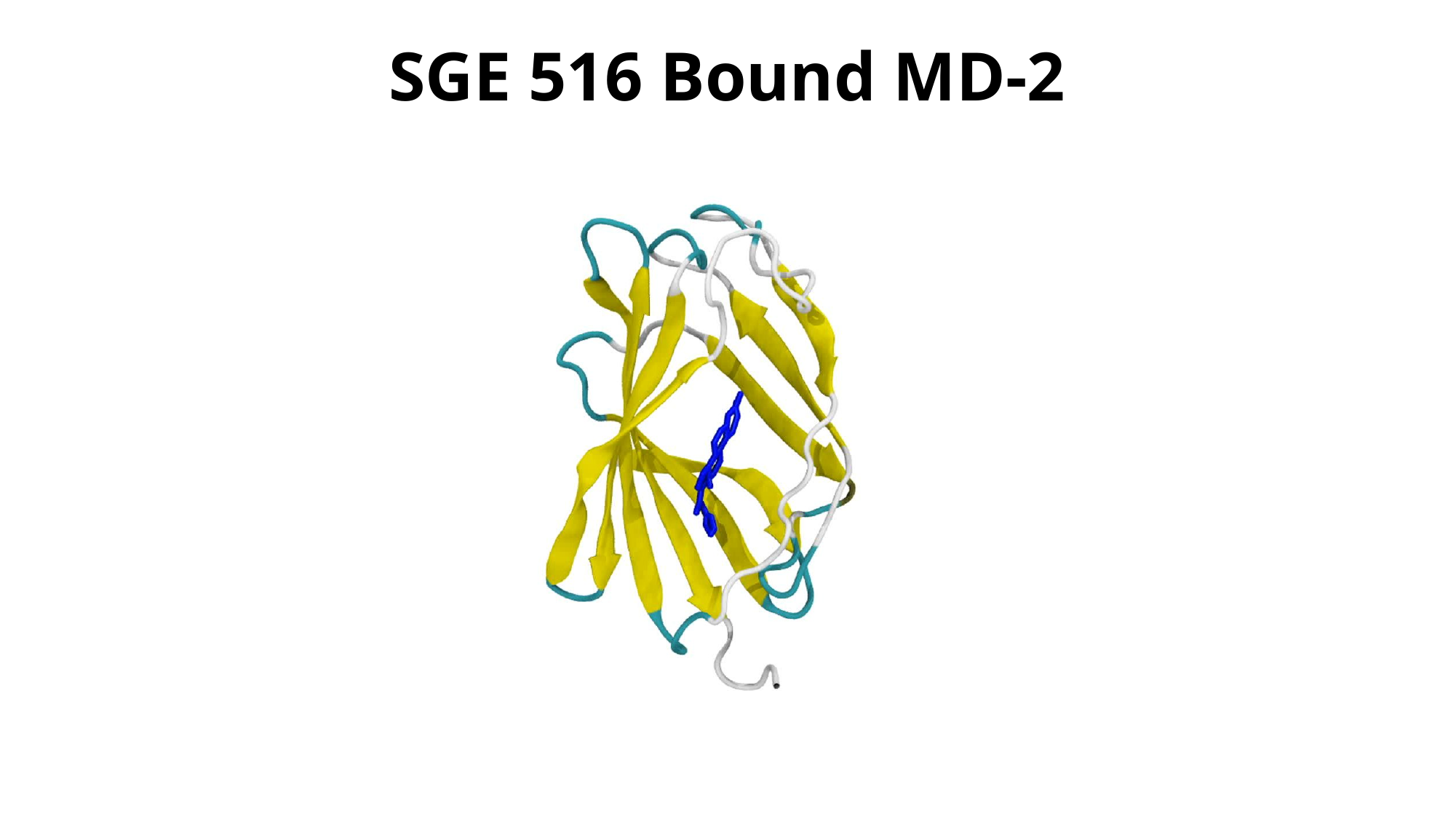

# SGE 516 Bound MD-2

Supplement: Supplementary file 2 [file Presentation2.pptx]

## Slide 1
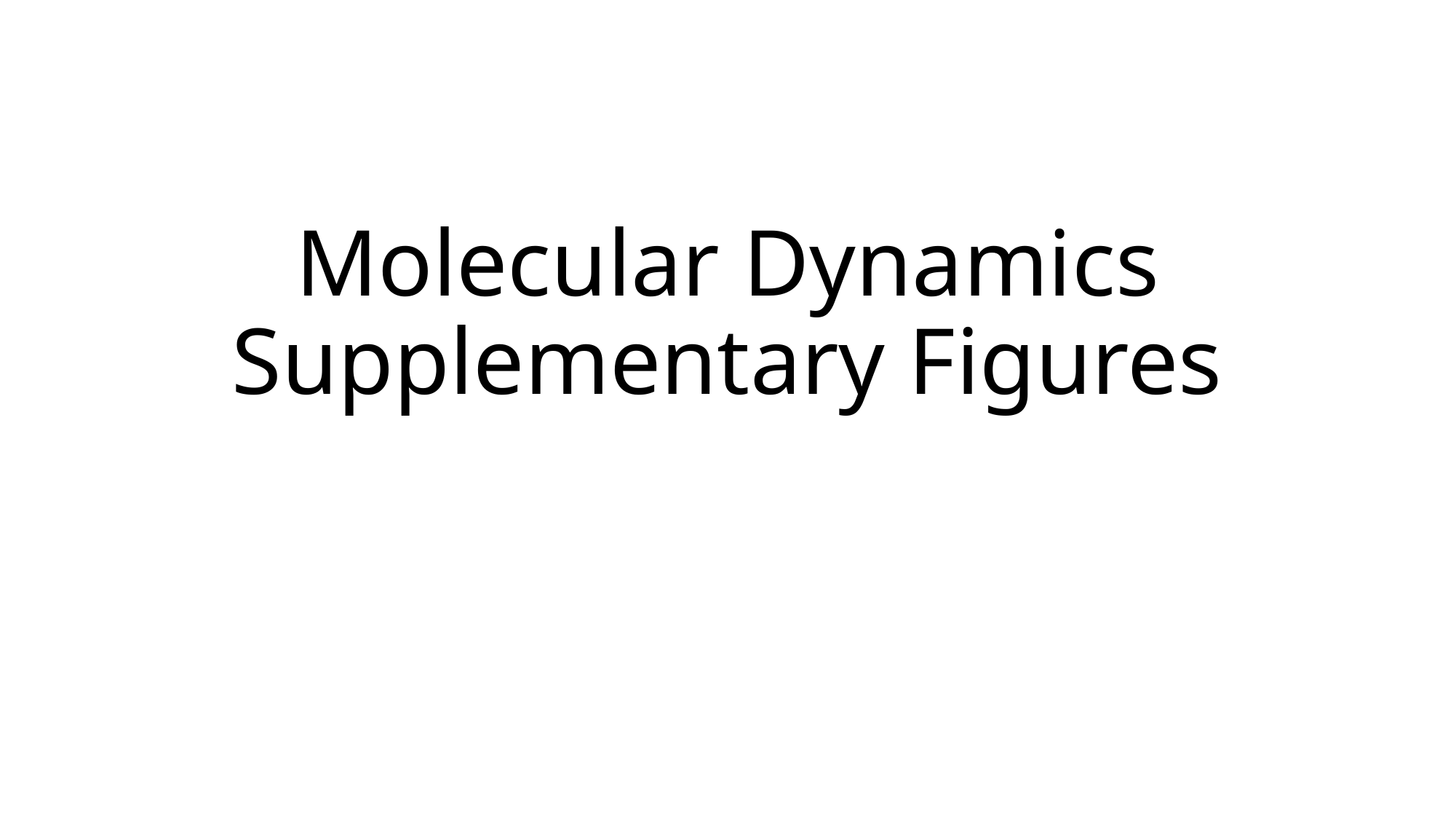

# Molecular Dynamics Supplementary Figures

## Slide 2
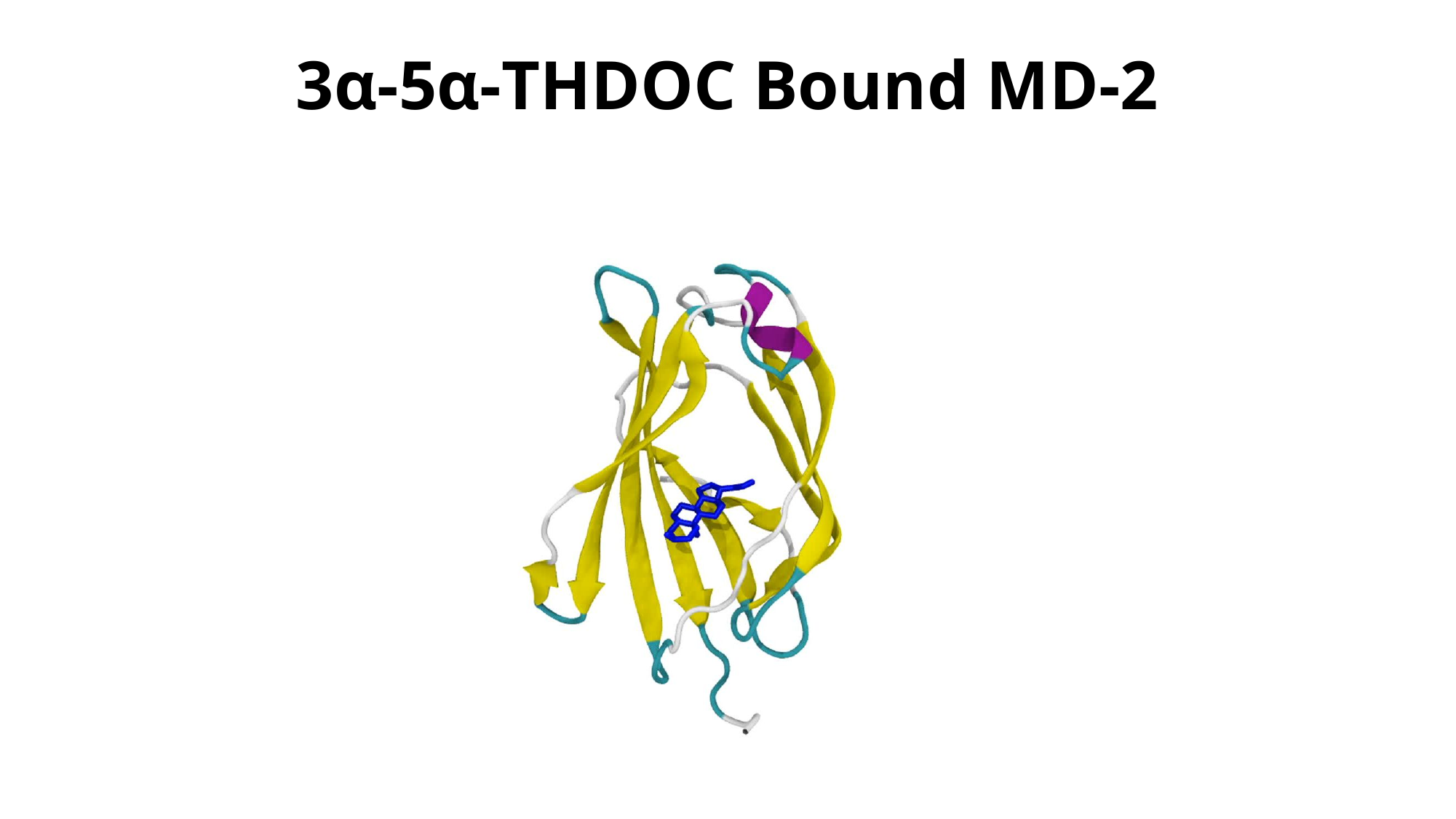

# 3α-5α-THDOC Bound MD-2

Supplement: Supplementary file 3 [file Presentation3.pptx]

## Slide 1
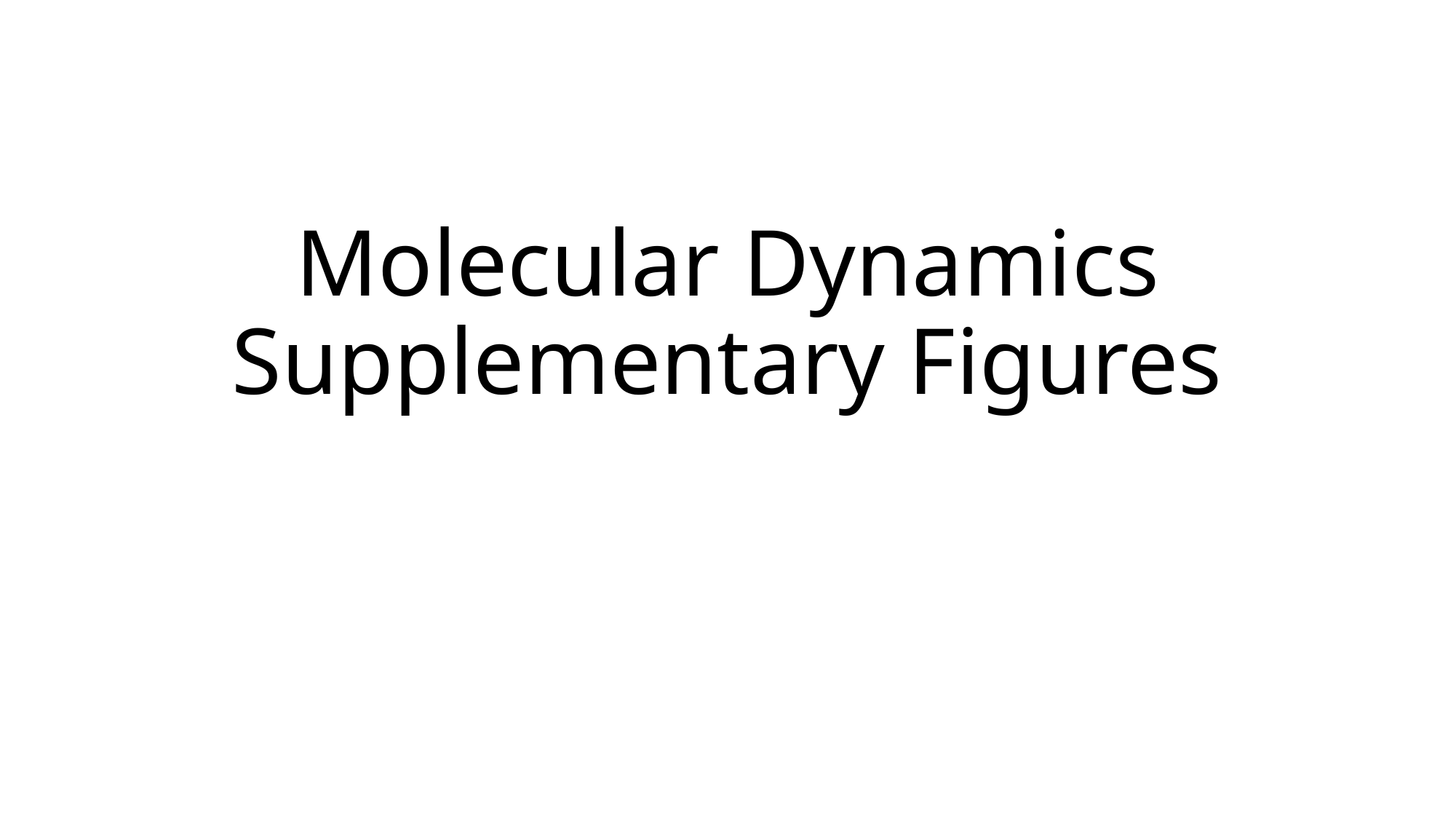

# Molecular Dynamics Supplementary Figures

## Slide 2
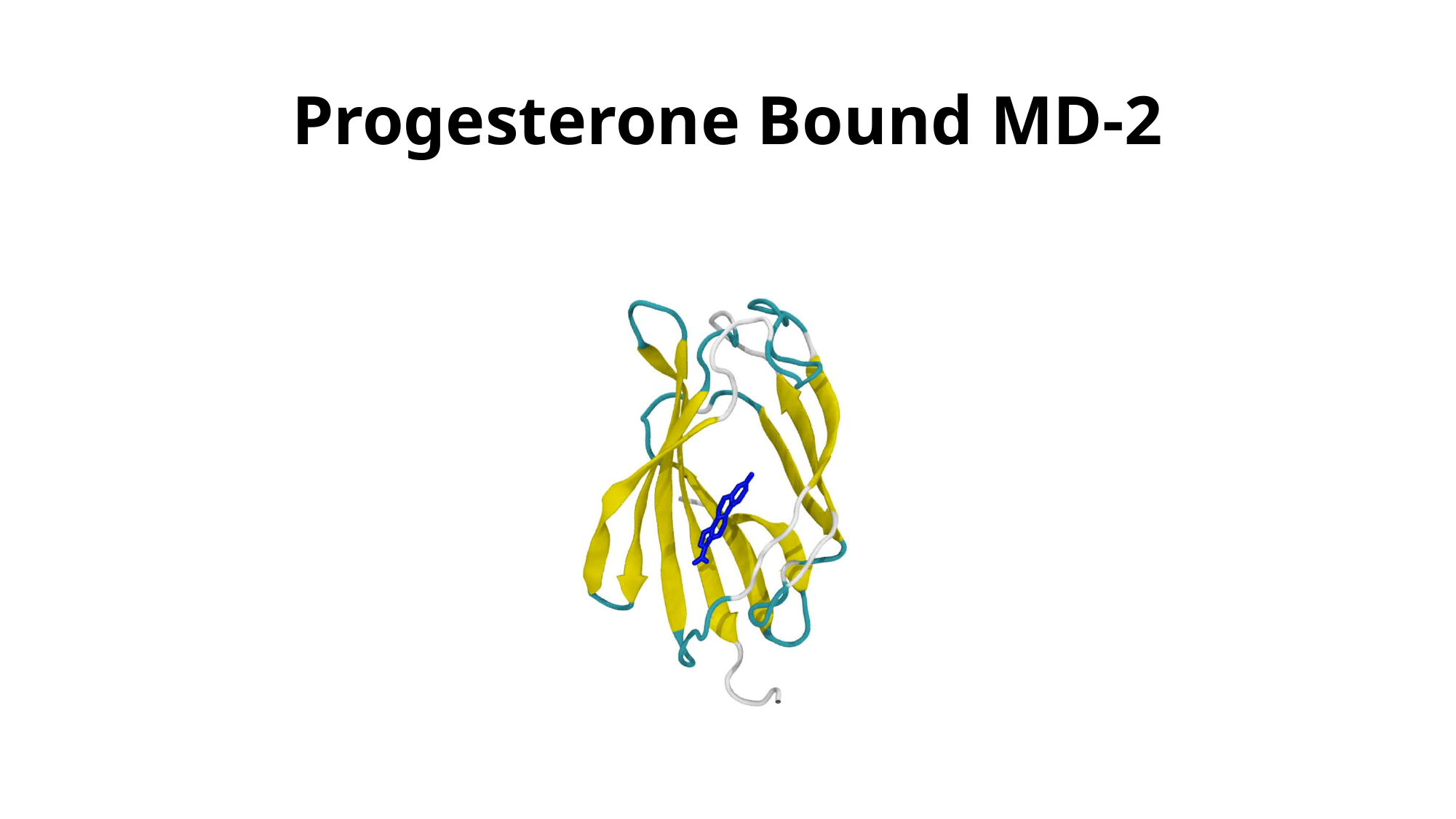

# Progesterone Bound MD-2

Supplement: Supplementary file 4 [file Presentation4.pptx]
